# Feedback circuitry via let-7c between lncRNA CCAT1 and c-Myc is involved in cigarette smoke extract-induced malignant transformation of HBE cells

**DOI:** 10.18632/oncotarget.15195

**Published:** 2017-02-08

**Authors:** Lu Lu, Hong Qi, Fei Luo, Hui Xu, Min Ling, Yu Qin, Ping Yang, Xinlu Liu, Qianlei Yang, Junchao Xue, Chao Chen, Jiachun Lu, Quanyong Xiang, Qizhan Liu, Qian Bian

**Affiliations:** ^1^ Institute of Toxicology, Ministry of Education, School of Public Health, Nanjing Medical University, Nanjing 211166, Jiangsu, People's Republic of China; ^2^ The Key Laboratory of Modern Toxicology, Ministry of Education, School of Public Health, Nanjing Medical University, Nanjing 211166, Jiangsu, People's Republic of China; ^3^ Jiangsu Center for Disease Control and Prevention, Nanjing 210009, Jiangsu, People's Republic China; ^4^ The School of Public Health, Institute for Chemical Carcinogenesis, Guangzhou Medical University, Guangzhou 510182, Guangdong, People's Republic China

**Keywords:** cigarette smoke extract (CSE), carcinogenesis, miRNAs, lncRNAs, lung cancer

## Abstract

Cigarette smoking is a primary risk factor for the development of lung cancer, which is regarded as the leading cause of cancer-related deaths. The process of malignant transformation of cells, however, is complex and elusive. The present study investigated the roles of an lncRNA, CCAT1, and a transcriptional factor, c-Myc, in human bronchial epithelial (HBE) cell transformation induced by cigarette smoke extract. With acute and chronic treatment of HBE cells, cigarette smoke extract induced increases of CCAT1 and c-Myc levels and decreases of levels of let-7c, a microRNA. Down-regulation of c-Myc reduced the degree of malignancy and the invasion/migration capacity of HBE cells transformed by cigarette smoke extract. ChIP assays established that c-Myc, increased by cigarette smoke extract, binds to the promoter of CCAT1, activating its transcription. Further, let-7c suppressed the expression of c-Myc through binding to its 3′-UTR. In turn, CCAT1 promoted the accumulation of c-Myc through binding to let-7c and decreasing free let-7c, which influenced the neoplastic capacity of HBE cells transformed by cigarette smoke extract. These results indicate that a positive feedback loop ensures expression of cigarette smoke extract-induced CCAT1 and c-Myc via let-7c, which is involved in cigarette smoke extract-induced malignant transformation of HBE cells. Thus, the present research establishes a new mechanism for the reciprocal regulation between CCAT1 and c-Myc and provides an understanding of cigarette smoke extract-induced lung carcinogenesis.

## INTRODUCTION

Although the globalization of tobacco use began centuries ago, the public health response to the consequences that it has caused is less than 50 years old [1]. Exposure to tobacco is primarily associated with cigarette smoke, which contains carcinogens that cause lung cancers [2, 3]. Lung cancer, an aggressive and heterogeneous disease caused mainly by smoking [4], results in more than 1 million deaths annually [5]. Although the association between lung cancer and cigarette smoke has been studied for several decades, the tumorigenic process caused by cigarette smoke remains largely unclear.

c-Myc, activated in various human malignancies, is an oncogenic transcription factor [6]. The c-Myc protein, often overexpressed in tumors, is essential for various cellular functions and is associated with poor cancer outcomes [7, 8]. In normal cells, the levels of c-Myc are strictly controlled; however, its deregulated expression results in uncontrolled cell proliferation and the formation of various cancers, including those of the lung [7]. Therefore, it is essential to understand the molecular networks involved, in particular, whether c-Myc participates in the tumorigenic process caused by cigarette smoke.

Many diseases are associated with an altered transcription pattern, which is not restricted to the production of aberrant levels of protein-coding RNAs, but includes dysregulation of the expression of noncoding members [9]. Although more than 90% of DNA is transcribed, most transcribed RNAs are noncoding [10–12]. Many are processed to generate small RNAs, including microRNAs (miRNAs), the best-known class of small RNAs [13, 14]. Other transcripts (long noncoding RNAs or lncRNAs) have, in their mature form, more than 200 nucleotides [15, 16]. Although the roles of miRNAs in the translation of protein-coding transcripts (mRNAs) have been studied, the influence of lncRNAs upon miRNA functions is only now being understood [17]. The interplay between lncRNAs and miRNAs during the tumorigenic process provides new insight into the regulatory mechanisms underlying noncoding RNA classes relevant to cancer [9]. The lncRNA, CCAT1 (colon cancer-associated transcript-1), also named CARLo-5 (cancer-associated region lncRNA-5), was first found to be upregulated in colon cancer [18]. CCAT1 is now known to be deregulated in various cancer types, including lung cancer [19–22]. Therefore, we propose that CCAT1 modulates the functions of miRNAs in cigarette smoke-induced carcinogenesis.

Although expression profiles for lncRNAs and transcription factors correlate with tumor growth [23–25], limited information is available regarding mechanisms by which alterations in lncRNAs and transcription factors contribute to cigarette smoke-induced carcinogenesis. In the present study, we investigated the relationship between CCAT1 and c-Myc in the tumorigenic process induced by cigarette smoke extract (CSE). The results reveal a feedback loop between CCAT1 and c-Myc, acting through let-7c, that promotes the CSE-induced tumorigenic process and presents a previously unknown mechanism by which CCAT1 and c-Myc contribute to CSE-induced carcinogenesis.

## RESULTS

### CSE increases the levels of c-Myc, affecting the malignancy of transformed HBE cells

**Figure 1:**
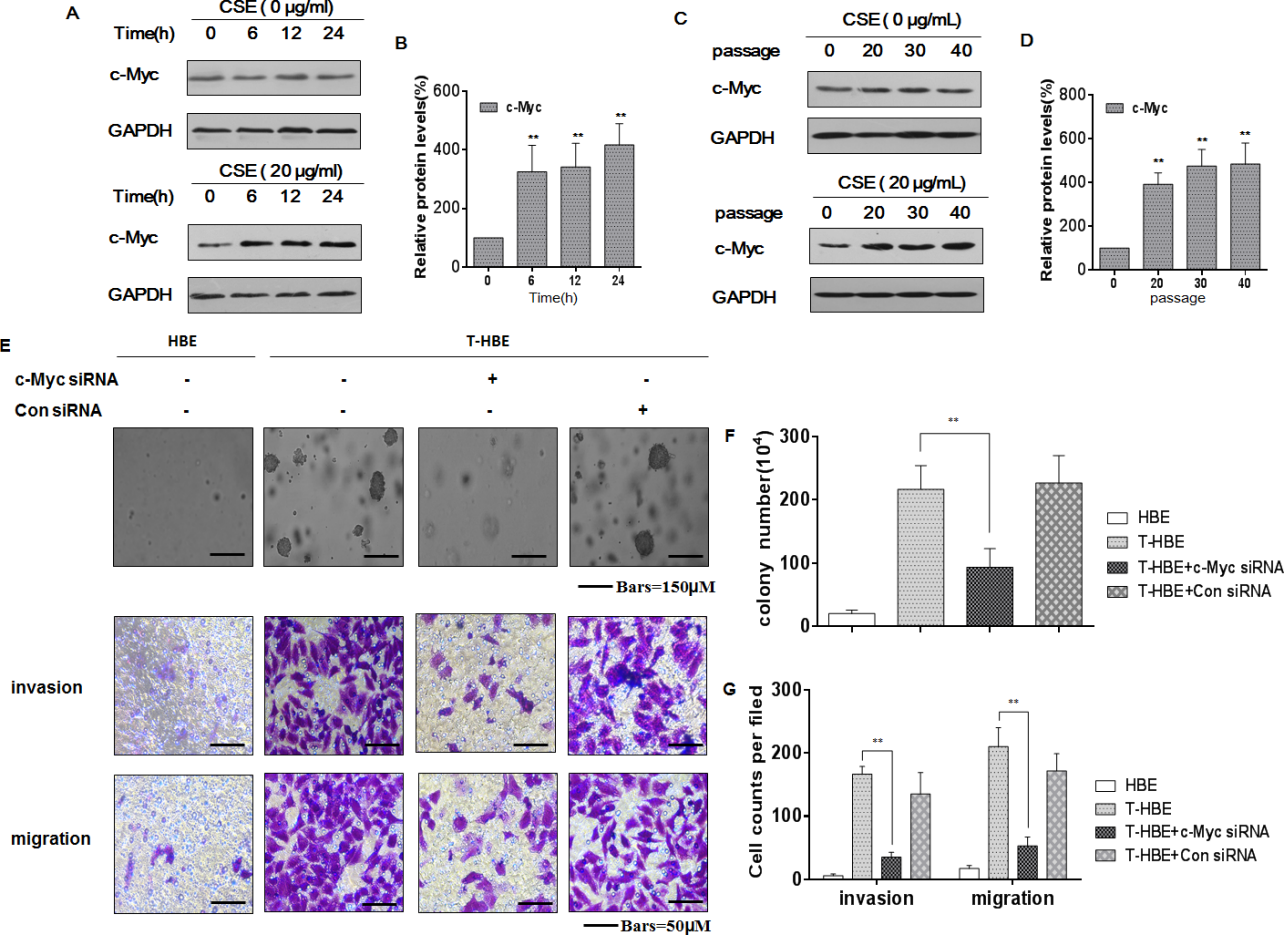
CSE increases the levels of c-Myc, affecting the degree of malignancy and the invasion/migration capacity of transformed HBE cells Abbreviations: HBE, passage-control HBE cells; T-HBE, CSE-transformed HBE cells. Densities of bands were quantified by Eagle Eye II software. GAPDH levels, measured in parallel, served as controls. HBE cells were exposed to CSE (0 or 20 μg/mL) for 0, 6, 12, or 24 h. (**A**) Western blots were performed, and (**B**) relative protein levels (means ± SD, *n* = 3) of c-Myc were determined. ** *P <* 0.05, different from control HBE cells. HBE cells were exposed to 0 or 20 μg/mL CSE for 0, 20, 30, or 40 passages. (**C**) Western blots were performed, and (**D**) relative protein levels (means ± SD, *n* = 3) of c-Myc were determined. *P < 0.05, different from passage-control HBE cells. T-HBE cells were transfected for 24 h with c-Myc siRNA or control siRNA at a final concentration of 100 ppm. (**E**) Representative images of colony formation in soft agar (upper, bars = 150 μm), cell invasion (middle, bars = 50 μm), and cell migration (lower, bars = 50 μm) were prepared. The numbers (means ± SD, *n* = 3) of colonies formed (**F**) and of invading or migrating cells (**G**) were quantified. **p < 0.05, different from T-HBE cells in the absence of c-Myc siRNA.

c-Myc, a transcription factor, is correlated with tumor aggression and poor clinical outcomes [26, 27]. In human malignancies, dysregulated expression or function of c-Myc is a common abnormality [28]. In the present study, we measured c-Myc levels in HBE cells exposed to 20 μg/mL CSE for 0, 6, 12, or 24 h or for 0, 20, 30, or 40 passages. With increased time of exposure to CSE in acute or chronic treatment of HBE cells, there was greater expression of c-Myc (Figure 1A–1D). Such changes were not evident in control cells. In assessing the function of cMyc in CSE-transformed cells, we found that, in the presence of c-Myc siRNA, the numbers of colonies and the invasion/migration capacities of CSE-transformed HBE cells were decreased (Figure 1E–1G). These results indicate that CSE induces increases of c-Myc levels, which enhance neoplastic activity in the CSE-induced malignant transformation of HBE cells.

### CSE induces increases of CCAT1 levels and decreases of let-7c levels in HBE cells

**Figure 2:**
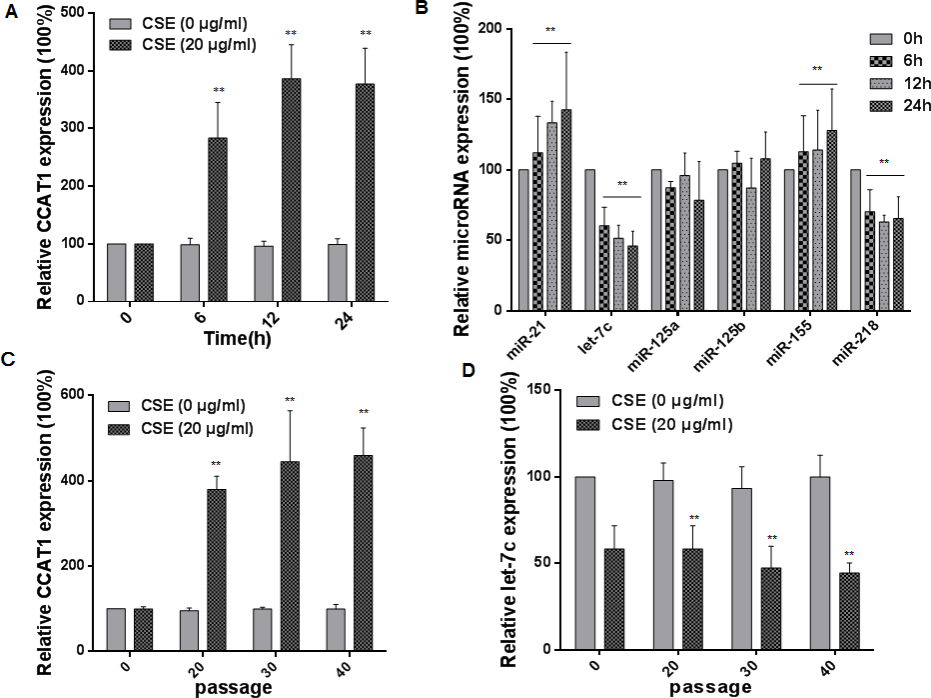
CSE induces increases of CCAT1 levels and decreases of let-7c levels in HBE cells HBE cells were exposed to CSE (0 or 20 μg/mL) for 0, 6, 12, or 24 h. The levels (means ± SD, *n* = 3) of CCAT1 (**A**) miR-21, let-7c, miR-125a, miR-125b, miR-155, and miR-218 (**B**) were determined by quantitative RT-PCR. ** *P <* 0.05, different from control HBE cells. HBE cells were exposed to 0 or 20 μg/mL CSE for 0, 20, 30, or 40 passages. The levels (means ± SD, *n* = 3) of CCAT1 (**C**) and let-7c (**D**) were determined by quantitative RT-PCR. **P < 0.05, different from passage-control HBE cells.

Various lncRNAs may function in tumor progression and metastasis [29, 30]. As shown in our previous research, exposure of cells to CSE affects levels of lncRNAs, and the lncRNA CCAT1 is related to the malignant characteristics of CSE transformed-HBE cells [31–33]. miRNAs can be used as biomarkers for exposure to environmental factors, including cigarette smoke, air pollution, nanoparticles, and diverse chemicals [34]. In the present study, we verified the expression of CCAT1 and measured various miRNAs associated with cigarette smoking in HBE cells exposed to 20 μg/mL CSE for 0, 6, 12, or 24 h. With longer times of exposure to CSE, there were greater expressions of CCAT1, miR-21, and miR-155 and lower expressions of let-7c and miR-218 (Figure 2A and 2B). Since the expression of let-7c was changed, and, in hepatocellular carcinomas and lung adenocarcinoma, CCAT1 promotes the proliferation and migration of cancer cells through functioning as a let-7 sponge [19, 35], we focused on CCAT1 and let-7c for further study. HBE cells were exposed to 0 or 20 μg/mL CSE for 0 to 40 passages. With longer times of exposure, there were increases of CCAT1 levels and decreases of let-7c levels (Figure 2C and 2D). Such changes were not found in control cells, indicating that their expressions were affected by CSE. These results show that, in HBE cells, CSE induces up-regulation of CCAT1 and down-regulation of let-7c.

### c-Myc increases CCAT1 expression via binding to the promoter of CCAT1 in HBE cells

**Figure 3:**
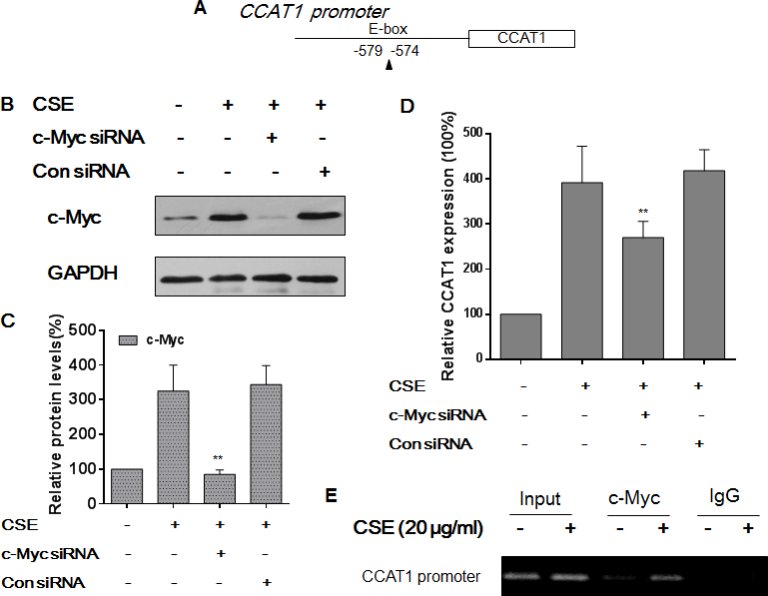
c-Myc regulates CCAT1 expression by binding to the promoter of CCAT1 in HBE cells Densities of bands were quantified by Eagle Eye II software. GAPDH levels, measured in parallel, served as controls. (**A**) Schematic graph illustrating binding sites of c-Myc in the promoter of CCAT1. HBE cells were exposed to CSE (0 or 20 μg/mL) in the absence or presence of 100 ppm c-Myc siRNA or control siRNA for 24 h. (**B**) Western blots and (**C**) relative protein levels (means ± SD, *n* = 3) of c-Myc were determined. (**D**) The levels (means ± SD, *n* = 3) of CCAT1 were determined by quantitative RT-PCR. **P < 0.05, different from CSE-treated HBE cells in the absence of the c-Myc siRNA. HBE cells were exposed to CSE (0 or 20 μg/mL) for 24 h. (**E**) the binding of c-Myc to promoters of CCAT1 was measured by a ChIP assay after chromatin was immunoprecipitated with an antibody against c-Myc.

Various transcription factors are involved in regulation of lncRNA transcription [15, 16]. To determine how transcription of CCAT1 is controlled, we searched for potential transcription factor binding sites in the promoter of CCAT1 (http://jaspar.genereg.net) and found one E-box element that could be recognized by c-Myc (Figure 3A). After they were transfected with c-Myc-specific siRNA or control siRNA for 24 h, HBE cells were exposed to CSE for 48 h. The transfection efficiency was assessed by Western blots (Figure 3B and 3C). After depletion of c-Myc, there were lower levels of CCAT1 compared with levels in cells exposed to CSE (Figure 3D). To explore the mechanism for c-Myc regulation of CCAT1, ChIP assays were performed for HBE cells exposed to CSE. For control and CSE-treated cells, the c-Myc antibody was used to immunoprecipitate chromatin-containing DNA fragments that included the promoter region of CCAT1. The results of ChIP and RT-PCR assays of HBE cells exposed to CSE showed that the amount of c-Myc binding to the promoter of CCAT1 was more than that in control HBE cells, which confirmed the interaction between c-Myc and the CCAT1 promoter (Figure 3E). These results indicate that, in CSE-treated HBE cells, c-Myc activates the expression of CCAT1 via binding to its promoter region.

### let-7c suppresses CSE-induced increases of c-Myc expression in HBE cells

**Figure 4:**
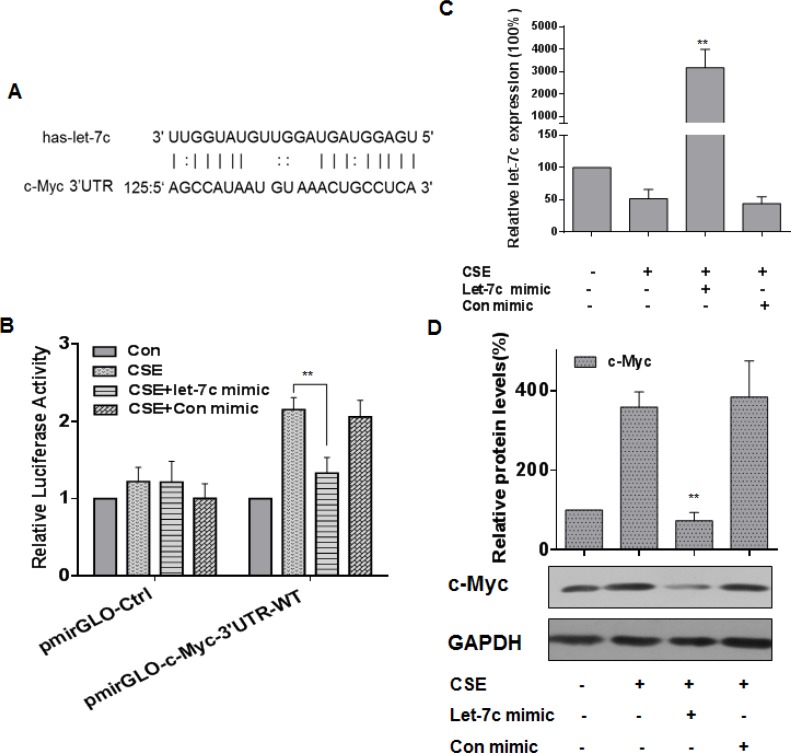
let-7c is involved in CSE-induced increases of c-Myc expression in HBE cells Densities of bands were quantified by Eagle Eye II software. GAPDH levels, measured in parallel, served as controls. (**A**) Schematic graph of the binding sites of let-7c in the 3′UTR of c-Myc. HBE cells were exposed to CSE (0 or 20 μg/mL) for 24 h after they were transfected with 50 nM let-7c mimic or control mimic for 24 h. HBE cells were co-transfected with pmirGLO-c-Myc-3′UTR -WT or pmirGLO-Ctrl and with 50 nM let-7c mimic or control mimic for 24 h, then exposed to CSE (0 or 20 μg/mL) for 24 h. (**B**) Luciferase activity was measured at 24 h after transfection. Means of triplicate assays with standard deviations were presented. (**C**) The levels (means ± SD, *n* = 3) of let-7c were determined by quantitative RT-PCR. **P < 0.05, different from CSE-treated HBE cells in the absence of the let-7c mimic. (**D**) Western blots and relative protein levels (means ± SD, *n* = 3) of c-Myc were determined. **P < 0.05, different from CSE-treated HBE cells in the absence of the let-7c mimic.

Various studies have shown that miRNAs, such as miR-155, let-7a, let-7c, and miR-145, regulate c-Myc expression [36–40]. Here, we used StarBase and microRNA.org (http://www.microrna.org/) to predict that let-7c forms complementary base pairing with c-Myc through binding to its 3′-UTR (Figure 4A). When the expression of let-7c was decreased in CSE-exposed HBE cells, the expression of c-Myc was increased (see Figure 1 and 2). To determine if let-7c inhibits c-Myc expression through binding to its 3′-UTR, luciferase reporter assays were conducted. The let-7c mimic reduced the luciferase activities of the cells co-transfected with pmirGLO-c-Myc-3′UTR-WT (Figure 4B). A let-7c mimic was transfected into HBE cells for 24 h, then the cells were exposed to CSE for 24 h. The transfection efficiency was assessed by quantitative RT-PCR (Figure 4C). Ectopic expression of let-7c attenuated the CSE-induced increase of c-Myc levels (Figure 4D). These results indicate that, in HBE cells, let-7c suppresses CSE-induced up-regulation of c-Myc.

### Through let-7c, CCAT1 increases the CSE-induced up-regulation of c-Myc expression in HBE cells

**Figure 5:**
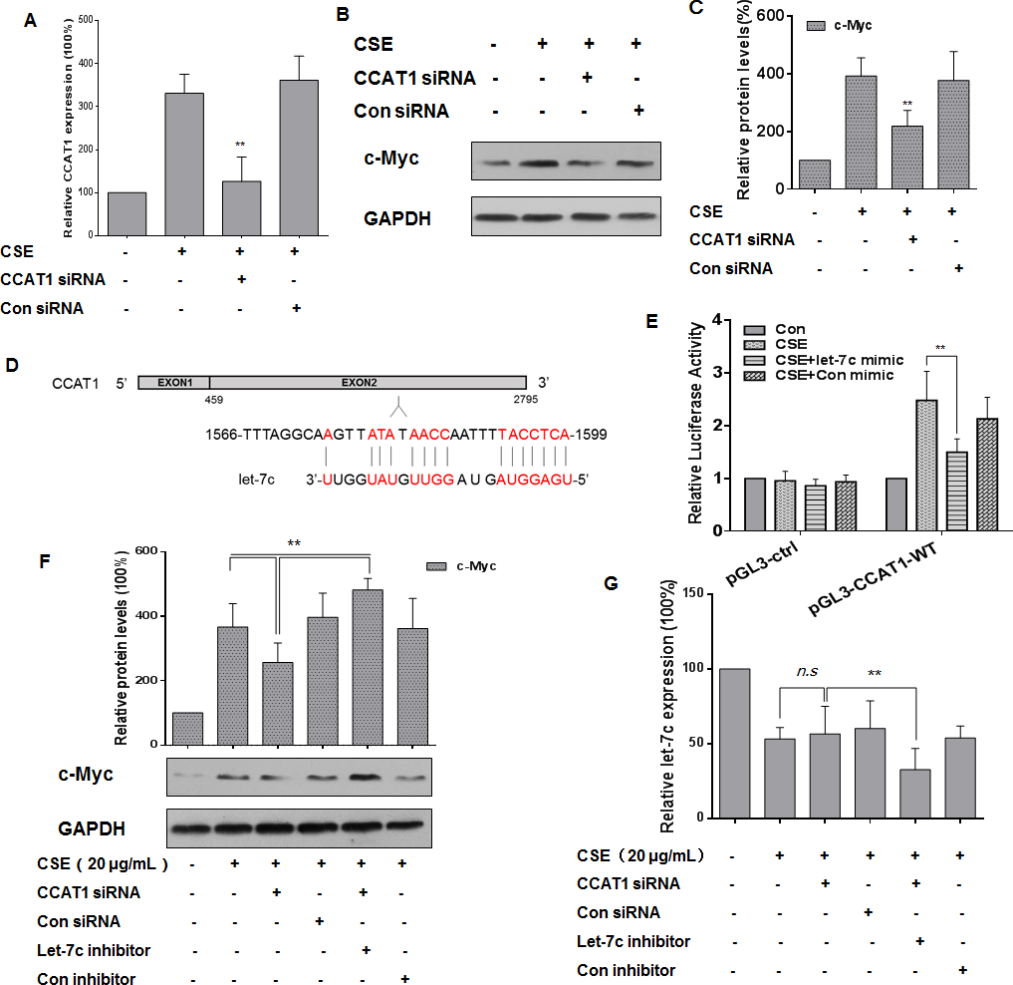
CCAT1 is involved in the CSE-induced elevation of c-Myc expression though let-7c in HBE cells Densities of bands were quantified by Eagle Eye II software. GAPDH levels, measured in parallel, served as controls. HBE cells were cultured in the presence of control siRNA or CCAT1 siRNA (100 ppm) for 24 h and then exposed to CSE (20 μg/mL) for 24 h. (**A**) The levels (means ± SD, *n* = 3) of CCAT1 were determined by quantitative RT-PCR. (**B**) Western blots and (**C**) relative protein levels (means ± SD, *n* = 3) of c-Myc were determined. **P < 0.05, different from CSE-treated HBE cells in the absence of CCAT1 siRNA. (**D**) Predicted binding sites for let-7c in CCAT1. HBE cells were co-transfected with pGL3-CCAT1-WT or pGL3-ctrl and with 50 nM let-7c mimic or control mimic for 24 h, then exposed to CSE (0 or 20 μg/mL) for 24 h. (**E**) Luciferase activity was measured at 24 h after transfection. Means of triplicate assays with standard deviations were presented. HBE cells were exposed to CSE (0 or 20 μg/mL) for 24 h after cells were co-transfected with CCAT1 siRNA and let-7c inhibitor for 24 h. (**F**) Western blots and relative protein levels (means ± SD, *n* = 3) of c-Myc were determined. **P < 0.05, different from CSE-treated HBE cells in the presence of CCAT1 siRNA. (**G**) The levels (means ± SD, *n* = 3) of let-7c were determined by quantitative RT-PCR. ** *P <* 0.05, different from CSE-treated HBE cells in the presence of CCAT1 siRNA. ‘n.s.’ indicates no significant difference from CSE-treated HBE cells in the presence of CCAT1 siRNA.

To investigate the relationship among CCAT1, let-7c, and c-Myc in CSE-treated HBE cells, these cells were treated with CCAT1 siRNA or control siRNA for 24 h, then exposed them to 0 or 20 μg/mL CSE for 24 h. The transfection efficiency was assessed by quantitative RT-PCR (Figure 5A). After CCAT1 silencing, CSE-induced increased levels of c-Myc were attenuated (Figure 5B and 5C). Since lncRNAs participate in regulation of the function of miRNAs [17, 41], we used the online software MiRCode (www.miRCode.org) and MicroInspector (http://bioinfo.uni-plovdiv.bg/ ) to predict that let-7c formed complementary base pairing with CCAT1 (Figure 5D). To confirm the binding between CCAT1 and let-7c, luciferase reporter assays were conducted. The wild-type CCAT1 reporter vector, together with a let-7c mimic or a control mimic, was transfected into HBE cells exposed to CSE (0 or 20 μg/mL). The let-7c mimic reduced the luciferase activities of the cells co-transfected with pGL3-CCAT1-WT (Figure 5E). As let-7c targets c-Myc through binding to its 3′-UTR (see Figure 4), we determined if the c-Myc levels were regulated by CCAT1 through let-7c. After depletion of CCAT1 and let-7c in CSE-exposed HBE cells, the expression of c-Myc was higher than that in cells treated with CCAT1 siRNA (Figure 5F). Relative to CSE-exposed HBE cells, there was no appreciable decrease of let-7c levels after down-regulation of CCAT1 (Figure 5G). The results indicate that CCAT1, at least in part, positively influences the CSE-induced increases of c-Myc levels through binding to let-7c.

### CCAT1, via let-7c, enhances the degree of malignancy and the invasion/migration capacity of CSE transformed-HBE cells

**Figure 6:**
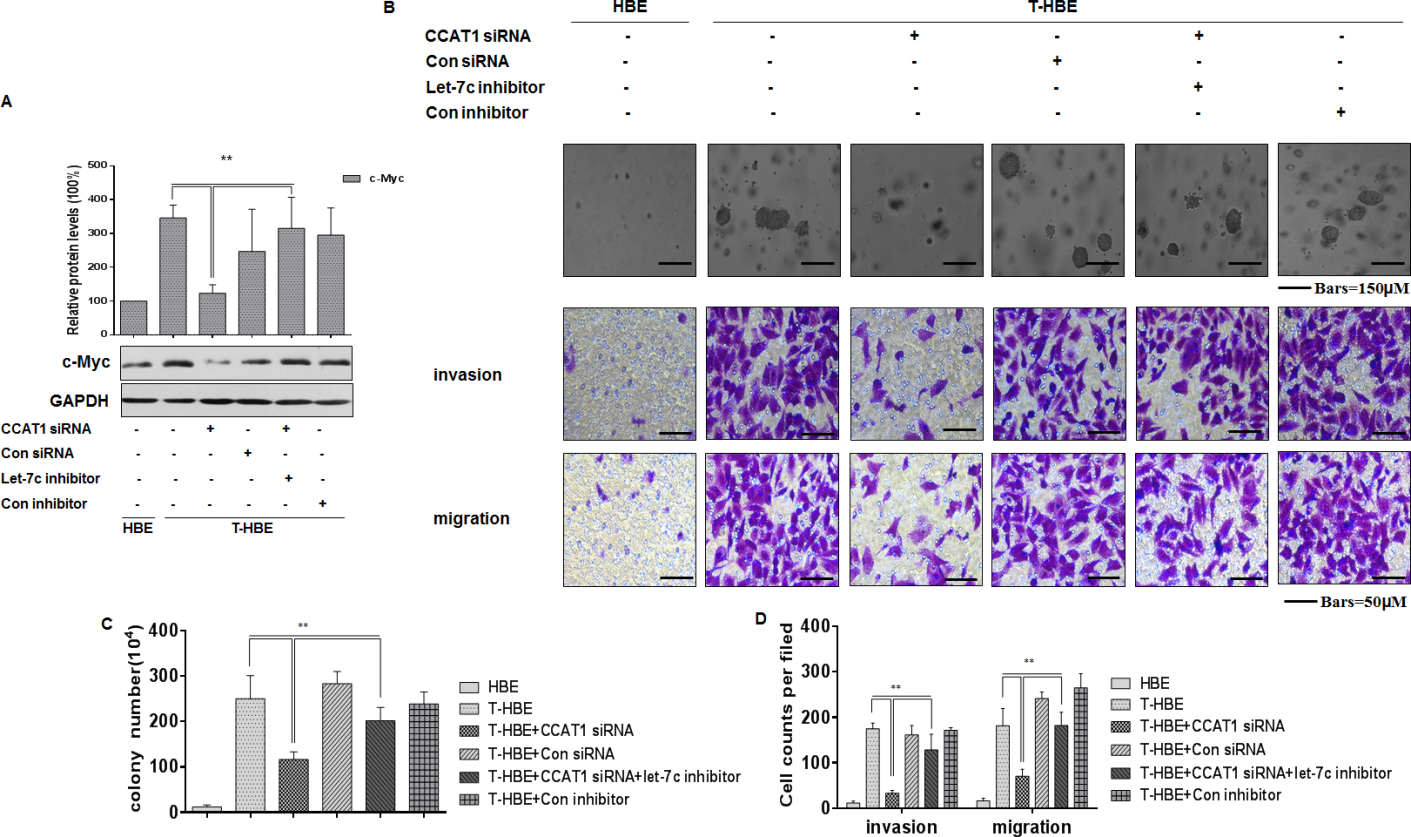
CCAT1, via let-7c, influences the degree of malignancy and the invasion/migration capacity of CSE transformed-HBE cells Abbreviations: HBE, passage-control HBE cells; T-HBE, CSE-transformed HBE cells. T-HBE cells were exposed to CCAT1 siRNA or control siRNA and to let-7c inhibitor or control inhibitor for 24 h. (**A**) Western blots and relative protein levels (means ± SD, *n* = 3) of c-Myc were determined. **P < 0.05, different from CSE-treated HBE cells in the presence of CCAT1 siRNA. (**B**) Representative images of colony formation in soft agar (upper, bars = 150 μm), cell invasion (middle, bars = 50 μm), and cell migration (lower, bars = 50 μm) were prepared. The numbers (means ± SD, *n* = 3) of colonies formed (**C**) and invading/migrating cells (**D**) were quantified. **P < 0.05, different from T-HBE cells in the presence of CCAT1 siRNA.

CCAT1, which is activated by the oncogene c-Myc, is involved in tumor development [22, 42, 43]. In the present study, we found that, in CSE-transformed HBE cells, the let-7c inhibitor reversed the CCAT1 siRNA-induced down-regulation of c-Myc (Figure 6A). And depletion of CCAT1 decreased colony formation and the invasion and migration capacities of CSE-transformed HBE cells; after co-transfection with CCAT1 siRNA and a let-7c inhibitor, these effects were reversed (Figure 6B–6D). These results establish that CCAT1 controls the degree of malignancy and the invasion/migration capacity of CSE transformed-HBE cells through regulating the function of let-7c.

**Figure 7:**
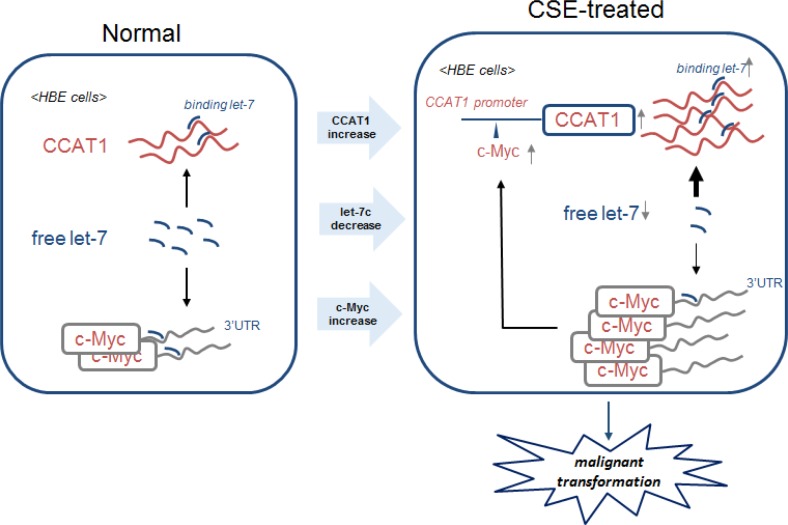
Schematic model of the feedback circuitry between c-Myc and CCAT1 acting via let-7c in CSE-induced transformation of HBE cells In HBE cells, CSE induces increases of CCAT1 levels and binding of let-7c and decreases of free let-7c levels, which causes increases of c-Myc. The enhancement of c-Myc promotes the expression of CCAT1 via binding to the promoter of CCAT1. The feedback circuitry, via let-7c between c-Myc and CCAT1, is involved in CSE-induced malignant transformation of HBE cells.

Further, in CSE-induced neoplastic transformation of HBE cells, over-expression of c-Myc increases CCAT1 expression through binding to its promoter region; in turn, CCAT1 increases c-Myc expression by binding free let-7c, which negatively regulates the expression of c-Myc through binding to its 3′-UTR (Figure 7). These results provide evidence that a positive feedback loop is formed to ensure CSE-induced CCAT1 and c-Myc expression, which are involved in transformation of cells and carcinogenesis.

## DISCUSSION

Lung cancer is a leading cause of death, and, in countries with high tobacco consumption, the proportion of cases attributable to smoking has reached up to 90% [4,44]. Cigarette smoke contains more than 4000 compounds, of which forty are carcinogenic [45]. Among these, nicotine, polycyclic aromatic hydrocarbons, and nicotine-derived nitrosamine ketone induce lung cancer through various signaling pathways [46–50]. In line with evaluations of individual compounds in cigarette smoke, studies with CSE have elucidated various signaling pathways and factors involved in the enhancing effect of cigarette smoke on lung cancer [31, 51–55]. Although cigarette smoke has effects on tumor growth and metastasis [45], the molecular events in carcinogenesis caused by cigarette smoke remain largely unclear.

c-Myc is a helix–loop–helix leucine zipper transcription factor that regulates an estimated 10–15% of genes in the human and Drosophila genomes [56]. As an oncoprotein, it regulates various cellular functions, such as cell division, growth, apoptosis, and differentiation. In human cancers, its expression is frequently elevated, and it is associated with tumor aggression and poor clinical outcomes [57]. In the present study, we found that the expression of c-Myc was increased in HBE cells exposed to CSE, and c-Myc silencing reduced the neoplastic capacity of CSE-transformed HBE cells. These results indicate that, in these cells, c-Myc acts as an oncogene. Further, we demonstrated the underlying mechanism by which c-Myc is involved in the CSE-induced transformation of HBE cells.

Most diseases, including human cancer, are associated with an altered transcription pattern [9]. lncRNAs, which are noncoding members with little or no protein-coding capability, function in a variety of biological processes and disease states [16, 58]. Moreover, deregulation of lncRNAs results in aberrant gene expression that contributes to the progression of cancers, including lung cancer [29, 59–62]. As confirmed in the present study, exposure of cells to CSE increases the expression of CCAT1, an oncogenic lncRNA [63]. CCAT1, acting via the transcription factor, c-Myc, is involved in pathogenesis of several malignancies, such as gastric carcinoma [22], hepatocellular carcinoma [20], and colorectal cancer [42, 43]. In the present study, we demonstrated that, in HBE cells, CSE-induced upregulation of c-Myc promotes CCAT1 expression through its binding to the promoter of CCAT1. Additionally, ChIP assays established that c-Myc, increased by CSE, binds to the promoter of CCAT1, indicating that c-Myc activates CCAT1 expression.

Acting by various mechanisms, most of which are repressive, miRNAs regulate the patterns of expressed proteins [64]. They regulate rates of gene transcription, inhibit the initiation and elongation of target mRNAs, promote decay of target mRNAs, and reduce the stability of proteins newly synthesized from target mRNAs [17, 34]. We investigated several miRNAs associated with cigarette smoking, including miR-21, let-7c, miR-125a, miR-125b, miR-155, and miR-218 [34]. In HBE cells, CSE induced a decrease of let-7c levels. The let-7 family is involved in the proliferation, apoptosis, and invasion of cancer cells [65]. The RNA-binding protein, HuR, inhibits c-Myc expression by recruiting let-7c-loaded RISC (RNA miRNA-induced silencing complex) to the c-Myc 3′-UTR [37]. In our study with HBE cells, a let-7c mimic inhibited the increase of c-Myc levels induced by CSE, which may have occur partly through binding to the c-Myc 3′-UTR.

The present results show that CCAT1 silencing reduces the increased expression of c-Myc caused by CSE. There is cross-regulation between miRNAs and lncRNAs [17], and lncRNAs affect the function of miRNAs [17]. It has been proposed that the concentration of miRNAs in the cytoplasm (and likely the nucleus) is titrated by abundant lncRNAs that harbor similar miRNA target sequences and hence sequester miRNAs away from mRNA [17, 66]. Since studies on the role of lncRNA-miRNA interactions in carcinogenesis are limited, we investigated the functions of CCAT1, along with those of miRNAs, which, in HBE cells, could contribute to the processes of malignant transformation caused by CSE. It has been reported that CCAT1 promotes the proliferation and migration of cancer cells depending on the sponging of let-7c [19, 35]. Here, we also found binding sites between CCAT1 and let-7c, and knockdown of CCAT1 did not appreciably change let-7c levels in CSE-treated cells, but, together with depletion of let-7c levels, the effect of CCAT1 silencing on the expression of c-Myc in CSE-exposed HBE cells was recovered, supporting the hypothesis that CCAT1 increases c-Myc expression though sponging of let-7c. We also demonstrated that, through binding let-7c, CCAT1 inhibits the function of let-7c, reducing c-Myc expression, which promotes proliferation and invasion/migration of CSE-transformed HBE cells. This supports the hypothesis that CCAT1 affects the expression of c-Myc, at least partially through regulating the function of let-7c in CSE-exposed HBE cells. The precise mechanism underlying the effect of cigarette smoke on lung cancer requires further investigation.

Thus, we have shown that CCAT1, an lncRNA, and c-Myc, a transcription factor, are over-expressed in the HBE cells exposed to CSE. Presenting further insight into the complex network underlying cigarette smoke-induced lung cancer, we have provided evidence for a previously unknown function for an lncRNA and a transcription factor in CSE-induced lung carcinogenesis. let-7c negatively regulates c-Myc, and CCAT1 increases c-Myc expression through sponging of let-7c. Our findings highlight the role of CCAT1 in regulation of the function of let-7c during exposure of HBE cells to CSE, which contributes to the overexpression of c-Myc. In turn, c-Myc transcriptionally activates CCAT1, thus forming a positive feedback loop to ensure CSE-induced CCAT1 and c-Myc expression, which are involved in malignant transformation of HBE cells. In sum, CCAT1 and c-Myc have a positive correlation, which expands understanding of the carcinogenic potential of cigarette smoke.

## MATERIALS AND METHODS

### Cells, cell culture methods, and reagents

Human bronchial epithelial (HBE) cells were obtained from the Shanghai Cell Bank of Chinese Academy of Sciences on 1th Jan 2014. Simian virus 40 (SV40)-transformed HBE cells are nontumorigenic and retain features of parent HBE cells. They are useful for studies of multistage bronchial epithelial carcinogenesis [67]. These cells were purchased from the Shanghai Institute of Cell Biology, Chinese Academy of Sciences (Shanghai, China). HBE cells were maintained in Eagle's minimum essential medium (MEM) with 10% fetal bovine serum (FBS, Life Technologies/Gibco, Grand Island, NY), 100 U/mL penicillin, and 100 μg/mL streptomycin (Life Technologies/Gibco, Gaithersburg, MD) and incubated in 5% CO_2_ at 37°C. At a concentration of 20 μg/mL, CSE does not change cell viability [54]. For chronic exposure, 1 × 10^6^ cells were seeded into 10-cm (diameter) dishes for 24 h and exposed to 0 or 20 μg/mL of CSE for 24–48 h per passage. This process was continued for 40 passages (about 20 weeks). CSE-transformed HBE (T-HBE) cells were those exposed to 20 μg/mL of CSE for 40 passages, during which they undergo malignant transformation [31, 54]. The cells in passage 0 (normal HBE cells) were not exposed to CSE. This process and the preparation of CSE were performed as described previously [54].

### Quantitative real-time PCR

Total RNA was isolated by use of TRIzol (Invitrogen) according to the manufacturer's instructions. Briefly, total RNA was prepared, and its concentration and integrity were assessed as previously described [54]. For detection of mature let-7c, 2 μg of total RNA, miRNA-specific stem-loop RT primers, and MMLV reverse transcriptase (Promega Corp., Madison, WI) were used in reverse transcription following the manufacturer's protocol. For measurement of CCAT1, the isolated RNA was reverse-transcribed into cDNA using a reverse transcription kit (Takara, Dalian, China). Quantitative real-time PCR was performed with an Applied Biosystems 7300HT machine and Maxima^TM^ SYBR Green/ROX qPCR Master Mix (Fermentas). U6 snRNA and 18 S RNA were used as internal controls to determine relative miRNA and lncRNA expressions. Relative gene expression was calculated by the formula 2^−(ΔΔCt)^ [68].

The following primers were used: *miR-21-F, 5*′*- ACACTCCAGCTGGGTAGCTTATCAGACTGA -3*′*, miR-21-R, 5*′*-TGGTGTCGTGGAGTCG-3*′*; let-7c-F, 5*′*- ACA CTCCAGCTGGGTGAGGTAGTAGGTTGT -3*′*, let-7c-R, 5*′*-TGGTGTCGTGGAGTCG-3*′*; miR-125a-F, 5*′*-ACACT CCAGCTGGGTCCCTGAGACCCTTTAAC-3*′*, miR-125a -R, 5*′*-TGGTGTCGTGGAGTCG-3*′*; miR-125b-F, 5*′*- ACACTCCAGCTGGGTCCCTGAGACCCTAAC-3*′*; miR-125b-R, 5*′*-TGGTGTCGTGGAGTCG-3*′*; miR-155-F, 5*′*- AC ACTCCAGCTGGGTTAATGCTAATCGTGAT-3*′*; miR-155- R, 5*′*-TGGTGTCGTGGAGTCG-3*′*; miR-218-F, 5*′*-ACAC TCCAGCTGGGTTGTGCTTGATCTAA-3*′*; miR-218-R, 5*′*-TGGTGTCGTGGAGTCG-3*′*; U6-F, 5*′*-CGCTTCGG CAGCACATATACTAAAATTGGAAC-3*′*; U6-R, 5*′*-GCT TCACGAATTTGCGTGTCATCCTTGC-3*′*; CCAT1-F, 5*′*-T TTATGCTTGAGCCTTGA-3*′*; CCAT1-R, 5*′*- CTTGCC TGAAATACTTGC-3*′*;* 18S-F, 5′*-GTAACCCGTTGAACC CCATT-3*′*;* and *18S-R, 5*′*- CCATCCAATCGGTAGTAG CG-3*′.

### Cell transfection

CCAT1 siRNA and control siRNA were purchased from Santa Cruz Biotechnology. The let-7c mimic/mimic control and the let-7c inhibitor/inhibitor control were purchased from Genechem, Shanghai, China. Cells were grown on six-well plates and transfected by use of Lipofectamine 2000 (Invitrogen), according to the manufacturer's instructions. At 24 h after transfection, cells were treated and subsequently harvested for qRT-PCR or Western blot analyses. All assays were performed in triplicate. The final concentrations employed were as follows: CCAT1 siRNA/negative control siRNA, 100 ppm; CCAT1-wt/CCAT1-ctrl, 50 nM; let-7c mimic/mimic control, 50 nM; and let-7c inhibitor/inhibitor control, 50 nM.

### Western blots

Western blot analyses to assess c-Myc and GADPH expression were accomplished as described previously [33]. Anti-glyceraldehyde 3-phosphate dehydrogenase (GAPDH) was purchased from Sigma, and an antibody for c-Myc was purchased from Abcam (Cambridge, MA). For densitometric analyses, the bands on the blots were measured by Eagle Eye II.

### Chromatin immunoprecipitation assays

Chromatin immunoprecipitation (ChIP) assays were performed by use of a Magna ChIP™ A/G Chromatin Immunoprecipitation Kit (Millipore) following the manufacturer's protocol. c-Myc antibody (Abcam, Cambridge, MA) and isotype IgG were used for immunoprecipitation. This process was performed as described previously [69].The specific sequences of immunoprecipitated and input DNA were determined by PCR primers for the CCAT1 promoter containing E-box: CCAT1 promoter forward, 5′-AGTCACTGGTGTTCTTGC-3′, and reverse, 5′-GGTATGCGTAGGTGATAGT-3′.

### Luciferase reporter gene assays

The 3′-UTR of c-Myc was sub-cloned into the luciferase reporter plasmid (pmirGLO-c-Myc-3′UTR-WT), and a 206-bp portion of CCAT1 (from 1491 nt to 1697 nt) was sub-cloned into a luciferase reporter plasmid (pGL3-CCAT1-WT), which together with a pmirGLO-Ctrl and pGL3-ctrl luciferase construct, were purchased from GeneChem (Shanghai, China). The pmirGLO-c-Myc-3′UTR-WT/pGL3-CCAT1-WT or pmirGLO-Ctrl/pGL3-ctrl was co-transfected with the let-7c mimic or mimic control into cells by Lipofectamine^®^ 2000 (Invitrogen)-mediated gene transfer according to the manufacturer's protocol. Approximately 24 h after transfection, the cells were washed three times with PBS (pH 7.4). Then the cells were lysed with 1× passive lysis buffer (Promega). The lysates from each well were analyzed in a 96-well plate illuminometer (Berthold Detection System, Pforzheim, Germany). The amounts of luciferase and Renilla luciferase were measured with the Dual-Luciferase Reporter Assay System kit (Promega) following the manufacturer's instructions. Transfection experiments were performed in quadruplicate and were repeated at least three times. The relative luciferase activity was normalized to Renilla luciferase activity, as reported previously [70].

### Colony formation assays

The cells were disassociated, suspended in MEM medium containing 0.35% agar, and plated onto 0.7% agar in triplicate at a density of 1 × 10^4^ cells/6-cm dish. The numbers of colonies that were > 0.5 mm in diameter were counted 14 days later, as reported previously [33].

### Transwell assays

The CSE-transformed HBE cells were suspended in serum-free medium at a density of 1 × 10^5^ cells/mL. Subsequently, 200 μL of cell suspensions were added to the upper chambers of Transwell plates with 8-μm pore membranes (Corning, Inc., Corning, NY, USA) with 35 μL of Matrigel (BD Biosciences, Franklin Lakes, NJ, USA). MEM media (500 μL) supplemented with 10% FBS was added to the lower chambers. After incubation for 24 h at 37°C, the cells on the upper surfaces of the microporous membranes were removed with cotton swabs, whereas cells on the lower surface of the membranes were fixed with 4% paraformaldehyde, stained with crystal violet solution for 30 min, and washed twice with PBS. Images of the stained cells from five selected views were captured under a microscope (high-power field), and the numbers of cells that migrated through the membranes were averaged. To assess the capacity for migration of transformed HBE cells, transfected cells (5 × 10^4^/100 μL) were added to upper chambers without Matrigel. MEM medium containing 10% FBS was added to the lower chambers. Cells were incubated for 24 h at 37°C. Migrating cells were fixed, stained, and calculated.

### Statistical analyses

All experiments were performed at least three times in triplicate for each group. The results are presented as means ± S.D. Comparisons of means among multiple groups were performed by one-way analysis of variance (ANOVA), and a multiple-range least significant difference (LSD) was used for inter-group comparisons. Statistical significance was reached if *P < 0.05*. All statistical analyses were performed with SPSS 16.0.
